# American Horses for the French Army

**Published:** 1896-03

**Authors:** M. J. Treacy

**Affiliations:** Veterinarian; 8th Cavalry


### ABSTRACTS FROM FOREIGN JOURNALS.

---

American Horses for the French Army. For the last two years the French Government has been purchasing many cavalry and artillery horses in this country, the principal market being at Indianapolis, Ind., which was selected, after visiting other horse centres, as being the best market for military animals.

The horses are purchased by a commission of army officers and an officer of the veterinary staff, and a horse is rejected if objected to by any member, but the veterinary officer's opinion is decisive on soundness, gait, and conformation.

The average price paid was $150, and the horses ranged from 15 to 16 hands and from 900 to 1150 pounds, the lighter horses for cavalry and the heavier ones for artillery. Stallions, geldings, and mares were purchased, preference being shown for mares, owing to their longevity, tractability, and other good and sufficient reasons, especially since they can be used as brood-mares when condemned as being useless for military work.

The veterinaay examination was very strict and thorough, and ten horses were rejected to every one purchased. Horses with either a long or sway back, or short pasterns, or vertical shoulders, were rejected at once, and all horses had to have a chest circumference at the eighth rib of 69 inches or over. Horses having large brand marks or excoriated lips, mouth, and ears were rejected, owing to the fact that they do not train well for the purposes required. In making a purchase the commission decided on the price to be paid, it was offered and, if accepted, the horse was branded with the government mark. No wrangling over the price was indulged in with the dealers.

This year the commission purchased 200 horses, shipping to France *via* Baltimore, their destination being the cavalry school at Saumur, where they are trained previous to being sent to their regiments. As to the gait, a regular walk, trot, and gallop were insisted upon ; no mixed gaits permitted. As to color, the horses were required to have a regular fixed color. No gray or piebald horses are purchased, nor those of a bleached color, the so-called "washy" horses.—*Veterinarian* M. J. Treacy, *8th Cavalry, in "Cavalry Journal."*

